# Retrospective isotope monitoring reveals spatial and temporal effects of anthropogenic pressures on the trophic ecology of European wildcats (*Felis silvestris*) in Germany

**DOI:** 10.1371/journal.pone.0343705

**Published:** 2026-02-25

**Authors:** Chris Baumann, Sabrina Streif, Ayenne S. Akarsu, Carsten Nowak, Dorothée G. Drucker

**Affiliations:** 1 AG Biogeology, Department of Geosciences, University of Tübingen, Tübingen, Germany; 2 Senckenberg Centre for Human Evolution and Palaeoenvironment (SHEP) at the University of Tübingen, Tübingen, Germany; 3 Forest Research Institute Baden-Württemberg, Freiburg, Germany; 4 Faculty of Biology and Psychology, University Göttingen, Göttingen, Germany; 5 Institute of Evolution and Ecology, Department of Biology, University of Tübingen, Tübingen, Germany; 6 Senckenberg Forschungsinstitut und Naturmuseum Frankfurt, Centre for Wildlife Genetics, Gelnhausen, Germany; Alaska Pacific University, UNITED STATES OF AMERICA

## Abstract

The European wildcat (*Felis silvestris*) is increasingly exposed to anthropogenic pressures, including habitat fragmentation, agricultural intensification, road mortality and hybridisation with domestic cats (*Felis catus*). These factors may alter trophic behaviour, ecological roles, and long-term conservation prospects. In this study, we use stable isotope analysis of cat hair (*δ*¹³C, *δ*¹⁵N, *δ*³⁴S) to assess dietary patterns and niche dynamics in wildcats, domestic cats, and their hybrids across three German regions. We combine two complementary case studies: (1) a spatial comparison between a core population *with low hybridisation rates* (Taunus) and a heavily introgressed range edge population (Markgräflerland), and (2) a 26-year retrospective dataset from Thuringia (East Thuringia, Hainich, Harz Foreland, Thuringian Basin, Thuringian Forest) to analyse temporal dietary trends and responses to landscape change. Our results reveal trophic differences among the taxa. Wildcats showed the narrowest isotopic niches, particularly in the Taunus, indicating strong ecological specialization. In contrast, hybrids occupied the broadest niches and showed substantial isotopic overlap with wildcats, especially in the region with high hybridisation rates. Domestic cats exhibited minimal niche overlap with wildcats, suggesting limited trophic competition. Long-term trends in Thuringian wildcats revealed increasing *δ*¹³C values over time, primarily in summer-grown hair, suggesting a growing reliance on prey associated with agricultural habitats. Correlations with land use and individual traits further highlighted how both factors shape isotopic signatures. Retrospective isotope monitoring using archived tissues provides a powerful, non-invasive tool to assess anthropogenic influences, hybridisation impacts, and long-term ecological change in elusive or protected carnivores such as the European wildcat.

## Introduction

The European wildcat (*Felis silvestris* [1]) is described as a solitary, crepuscular, and territorial species that tends to avoid humans [2,3]. However, wildcat populations have been expanding in Europe in recent years and are increasingly entering areas used by humans [4]. Stray, feral, and free-ranging domestic cats (*Felis catus* [1]) can come into close contact with wildcats and may influence their ecology, behaviour and population, as genetic studies have shown [5–9]. Hybridization of European wildcats with domestic cats is considered widespread and could lead to the cryptic extinction of some wild populations [3,10]. Especially in areas of population expansion, such as in Germany [2], an increased risk of hybridization is expected due to an overall observation that range increase can foster hybridization rates, which may be even elevated within anthropogenic landscapes with high human and domestic cat densities [2,4]. Elevated hybridization rates have been detected in various European regions [11,12] and recently also within Germany [13]. Moreover, an increasing contact between wildcats and domestic cats may pose an increased risk of disease infection, which is considered as an additional human caused threat. All pathogens of infectious diseases relevant to domestic cats such as *Feline Immunodeficiency Virus* (FIV) and *Feline Leukemia Virus* (FeLV) already occur in the German wildcat population [14]. Additionally, the European wildcat is not only threatened by the impact of and competition with domestic cats, but also by several other anthropogenic factors, including habitat fragmentation and destruction, as well as human-induced mortality (e.g., hunting, poisoning, traffic) [2,15,16].

Despite all these restrictions and threats, the European wildcat continues to spread, at least in its central European range. The observed wildcat expansion could be related to the ongoing anthropogenic climate change [17], as the wildcat is a temperate-adapted species. In addition, strict legal protection implemented in Germany since 1935 has likely supported population recovery and gradual recolonisation [14]. At the same time, wildcats may have adapted to human-made environments through a synanthropic behaviour. Synanthropes are defined as wild animals that benefit from a shared ecology with humans. Broadly, they can, for example, benefit from increased access to stable, food sources (e.g., via increased densities of prey resulting from urbanization or agriculture), and from a reduction of predation pressures within human-altered habitats [18]. Among the most well-known animals that have followed this path are the house mouse (*Mus musculus* [19,20]), the African wildcat (*Felis lybica* [21–26]), and the red fox (*Vulpes vulpes* [27,28]).

**Fig 1:**
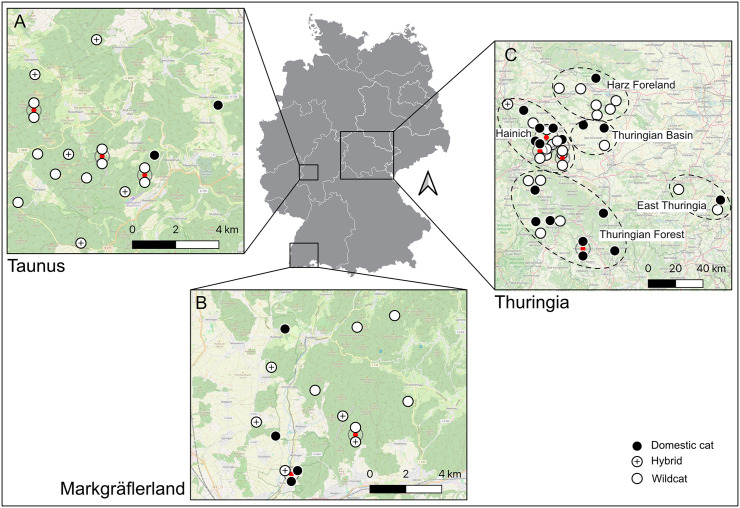
Map of the study area showing the locations of both case studies. Region A (Taunus) and Region B (Markgräflerland) represent the first case study, which is based on cat hair samples collected from lure stick monitoring in spring 2021. Region C (Thuringia) corresponds to the second case study, involving hair samples from road-killed wildcats collected between 1995 and 2021. Map data from OpenStreetMap (CC BY-SA 2.0).

To understand the factors driving the range expansion of the European wildcat and its ecological responses to competition with its domestic congener, Germany represents a particularly suitable study area (Fig 1). In recent decades, wildcat populations have increased markedly in Germany, and individuals are observed more and more frequently in open habitats near human settlements, where hybridization with domestic cats can occur [2,4,29–31]. According to data from 2024, estimations by national conservation organizations suggest that approximately 6,000 wildcats inhabit Germany (https://www.deutschewildtierstiftung.de/wildtiere/wildkatze). The combination of intensively managed forests, a dense road network, and a high abundance of domestic cats creates conditions that promote ecological interactions and potential competition between the two cat species.

To gain insights into the ecology of the wildcat, including its adaptation, competition, and range expansion, understanding its diet is essential. Trophic ecology is often a key factor in niche differentiation, species distribution, and interspecific competition [32–35]. A common approach to studying the feeding ecology of wildcats involves stomach content analysis from dead individuals [33,36]. While this method provides detailed insights into the animal’s last meal, it requires large sample sizes of carcasses and dissected stomachs to detect trophic patterns over extended time periods. To overcome this limitation, scat analysis is often used in wildcat habitats to obtain a more time-integrated view of dietary habits [37–39]. However, this method also has limitations, particularly in sample availability. Unlike canids or mustelids, felids tend to bury their faeces and do not use them as territorial markers, making scat samples difficult to locate. In this study, we therefore focus on a more robust and widely applied alternative in other regions of the world: stable isotope analysis of hair samples and retrospective isotope monitoring [40].

One major advantage of this method is that it often does not require additional sampling efforts, as suitable material is already available in natural history museums and scientific collections. In the case of wildcats in Germany, for example, hair samples are regularly collected in the frame of the nationwide genetic monitoring programs or regional wildcat assessments using lure stick-based hair trapping [41]. Access to these archived materials allows for a non-invasive investigation of wildcat diet through stable isotope analysis. Furthermore, this approach highlights the scientific value of natural history collections, which, often assembled over decades, can be revisited with modern analytical tools to address contemporary ecological questions.

### Stable isotope tracking

Stable isotope analysis is a well-established tool in (paleo-)ecological research, used to infer trophic relationships and dietary patterns over time (e.g., [21,28,42–44]). By analysing the ratios of naturally occurring stable isotopes, most commonly carbon and nitrogen, and sulfur, in biological tissues, it is possible to reconstruct feeding ecology over extended periods, as, e.g., hair integrate isotopic signals during its growth [44]. Carbon isotopes (*δ*¹³C) provide information on the primary sources of dietary carbon and are useful for distinguishing between prey from different habitats or consuming plants using different photosynthetic pathways (e.g., forest vs. open-field species, C₃ vs. C₄ plants) [40,42,45,46]. Nitrogen isotopes (*δ*¹⁵N) reflect an organism’s trophic position, with higher *δ*¹⁵N values indicating higher positions in the food web [40,47]. Sulfur isotopes (*δ*³⁴S) can help differentiate between terrestrial and aquatic food sources, or can be used as regional tracking isotope [40,48,49]. It is important to note that these isotopic signals are primarily incorporated from the protein fraction of the diet; therefore, stable isotope analysis from hair allows for the reconstruction of protein sources rather than the complete dietary composition. Interpreting isotope values in carnivores, however, is more challenging than in herbivores, as carnivores typically consume a variety of prey species that may come from different habitats and trophic levels. While herbivore isotope signatures are closely tied to local vegetation and thus reflect habitat use more directly, carnivore isotopic profiles represent an averaged signal of their prey’s isotopic values. This complexity necessitates cautious interpretation; however, stable isotope analysis can still reveal meaningful ecological patterns, such as preferred hunting grounds or dietary specialization and overlap [50,51].

**Fig 2:**
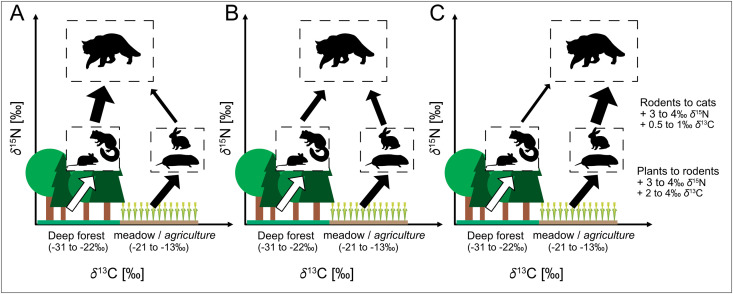
Schematic representation of potential trophic strategies of the European wildcat. **A)** Primary prey (small mammals) derived from forest habitats, B) mixed diet including small mammals from both forest and open land habitats, C) prey mainly derived from open land habitats. Values in brackets indicate the typical δ¹³C range of deep forest and meadow/agriculture areas.

In the case of European wildcats, stable isotope analysis can offer valuable insights into both habitat use and dietary flexibility (Fig 2). *δ*¹³C values may indicate whether individuals predominantly hunt in forested areas, open landscapes, or agricultural zones, based on the habitat affiliation of their prey. *δ*¹⁵N values can reveal trophic shifts, such as a reliance on rodents and small vertebrates versus the inclusion of anthropogenic food sources. Elevated *δ*¹⁵N values may arise either from prey that themselves feed on human-derived resources (e.g., commensal rodents in agricultural or urban environments) or from the direct consumption of anthropogenic material such as food waste or pet food. Finally, *δ*³⁴S values may help detect the consumption of aquatic or marine-derived resources; for instance, the presence of marine sulfur signatures in inland wildcats would strongly suggest access to human-related food sources such as food waste or pet food.

### Aim and case studies

This study aims to evaluate the potential of retrospective stable isotope monitoring to investigate anthropogenic influences on the trophic ecology of European wildcats. By integrating two complementary case studies, we explore how human-induced factors, such as hybridization, landscape modification, and agricultural practices, shape wildcat foraging behaviour over space and time. Our overarching goal is to demonstrate how stable isotope data from hair can be used to reconstruct dietary patterns, assess niche dynamics, and identify drivers of ecological change in carnivores.

The first case study is based on hair samples (n = 31) collected in spring 2021 using the lure stick method in two regions of Germany (Fig 1A & B): the Markgräflerland in Baden-Württemberg and the Taunus region in Hesse. These samples were originally collected in the frame of the national wildcat monitoring program. The two populations differ markedly in their conservation histories: the Taunus population represents a stable, long-established wildcat population with a low hybridization rate (~2%), whereas the Markgräflerland population is the result of recent natural recolonization from neighbouring French populations and exhibits a much higher hybridization rate (~43%) [30]. Against this background, we investigated how wildcats, hybrids, and domestic cats partition trophic space within each region, and to what extent hybrids and domestic cats overlap with wildcats under contrasting hybridisation regimes, thereby evaluating hybridization as a potential anthropogenic driver of trophic change.

The second case study focuses on 47 hair samples from deceased wildcats and wildcat-like domestic cats collected between 1995 and 2021 across various regions of Thuringia (Fig 1C), sourced from the Phyletic Museum in Jena. These regions encompass a broad gradient of landscape types, ranging from predominantly forested and structurally complex landscapes (Thuringian Forest, Hainich), to agriculturally intensive lowland areas with a high proportion of arable land and comparatively low forest cover (Thuringian Basin, Harz Foreland), as well as more heterogeneous forest–field mosaics characterized by mixed land use, smaller field sizes, and a higher density of edges and ecotones (East Thuringia). Through stable isotope analysis of carbon, nitrogen, and sulfur, we reconstructed temporal dietary trends and investigated how wildcat foraging behaviour may have shifted over time in response to regional climate change, agricultural development, and other landscape-level changes. We also assessed relationships between trophic variation and individual traits such as body size and sex.

Together, these case studies provide a framework to assess the trophic responses of wildcats to multiple dimensions of anthropogenic pressure.

## Results

Out of the initial 78 cat hair samples, five were excluded due to atomic C/N ratios falling outside the acceptable range (3.0 to 4.05), and one additional sample were removed due to excessively high carbon (%C > 50) content. The remaining 72 samples were retained for stable isotope analysis. A complete list of raw isotopic data is provided in S1 Table. Summary statistics of the isotopic values grouped by taxon and region are presented in Table 1. Most data were available for wildcats (n = 38), followed by domestic cats (n = 23) and hybrids (n = 11). Regionally, the majority of samples originated from the Hainich and Taunus regions (18 and 16 individuals, respectively), while smaller sample sizes were available for East Thuringia, Harz Foreland, Markgräflerland, Thuringian Basin, and Thuringian Forest (ranging from 3 to 12 individuals per region).

**Table 1:** Summary of stable isotope values (δ¹³C, δ¹⁵N, δ³⁴S) from cat hair, grouped by taxon and region.

Taxon	n	Region	*Case study*	*δ*¹³C_cor_ (mean ± SD)	*δ*¹³C_cor_ min	*δ*¹³C_cor_ max	*δ*¹⁵N (mean ± SD)	*δ*¹⁵N min	*δ*¹⁵N max	*δ*³⁴S (mean ± SD)	*δ*³⁴S min	*δ*³⁴S max
Domestic Cat	23	all samples	1 & 2	−19.8 ± 1.3	−22.7	−17.3	6.0 ± 1.5	4.0	10.8	5.9 ± 1.3	3.5	11.0
Hybrid	11	all samples	1 & 2	−19.7 ± 1.7	−24.0	−17.5	4.4 ± 2.3	1.6	9.1	3.7 ± 0.7	2.5	4.7
Wildcat	38	all samples	1 & 2	−20.5 ± 1.4	−22.8	−17.7	3.6 ± 1.7	0.6	6.7	4.4 ± 1.1	2.4	6.3
Domestic Cat	4	Markgräflerland	1	−19.5 ± 2.0	−22.2	−17.7	6.7 ± 0.9	5.6	7.8	4.8 ± 1.0	3.5	5.9
Hybrid	5	Markgräflerland	1	−19.5 ± 0.9	−20.2	−18.1	5.0 ± 2.7	1.9	9.1	3.8 ± 0.8	2.7	4.7
Wildcat	5	Markgräflerland	1	−19.9 ± 0.6	−20.6	−19.2	3.7 ± 1.2	2.4	5.1	3.6 ± 0.9	2.4	4.9
Domestic Cat	1	Taunus	1	−19.9	−19.9	−19.9	5.8	5.8	5.8	4.9	4.9	4.9
Hybrid	5	Taunus	1	−19.1 ± 1.2	−20.3	−17.5	3.5 ± 1.7	1.6	5.5	3.6 ± 0.6	2.5	3.9
Wildcat	10	Taunus	1	−19.2 ± 0.9	−20.4	−17.7	3.0 ± 1.1	1.5	5.3	3.4 ± 0.3	2.9	3.9
Domestic Cat	1	East Thuringia	2	−20.2	−20.2	−20.2	6.0	6.0	6.0	6.5	6.5	6.5
Wildcat	2	East Thuringia	2	−20.9 ± 1.4	−21.8	−19.9	3.6 ± 4.1	0.7	6.5	4.6 ± 1.0	3.9	5.3
Domestic Cat	7	Hainich	2	−20.0 ± 1.6	−22.5	−19.6	6.1 ± 2.3	4.2	10.8	6.4 ± 2.1	4.8	11.0
Hybrid	1	Hainich	2	−24.0	−24.0	−24.0	6.6	6.6	6.6	4.2	4.2	4.2
Wildcat	10	Hainich	2	−21.0 ± 1.2	−22.8	−19.1	3.6 ± 1.8	0.7	5.9	5.2 ± 0.7	3.8	6.3
Domestic Cat	1	Harz Foreland	2	−18.9	−18.9	−18.9	5.6	5.6	5.6	5.8	5.8	5.8
Wildcat	6	Harz Foreland	2	−20.9 ± 1.1	−22.5	−19.6	3.2 ± 2.0	0.6	5.7	4.7 ± 1.0	3.6	6.0
Domestic Cat	2	Thuringian Basin	2	−20.2 ± 0.1	−20.3	−20.1	5.4 ± 2.1	4.0	6.9	6.1 ± 0.7	5.7	6.6
Wildcat	1	Thuringian Basin	2	−22.2	−22.2	−22.2	6.7	6.7	6.7	5.8	5.8	5.8
Domestic Cat	7	Thuringian Forest	2	−19.9 ± 1.3	−21.2	−17.3	5.7 ± 1.0	4.5	7.3	6.1 ± 0.3‰	5.8	6.5
Wildcat	4	Thuringian Forest	2	−21.9 ± 0.9	−22.7	−20.6	4.6 ± 1.9	2.5	6.7	4.8 ± 1.0	3.5	6.1

δ¹³C values are lipid and Suess-effect corrected. For each group, the number of individuals (n), arithmetic mean ± standard deviation (SD), as well as minimum and maximum values are reported. Isotopic values are given in ‰.

**Fig 3:**
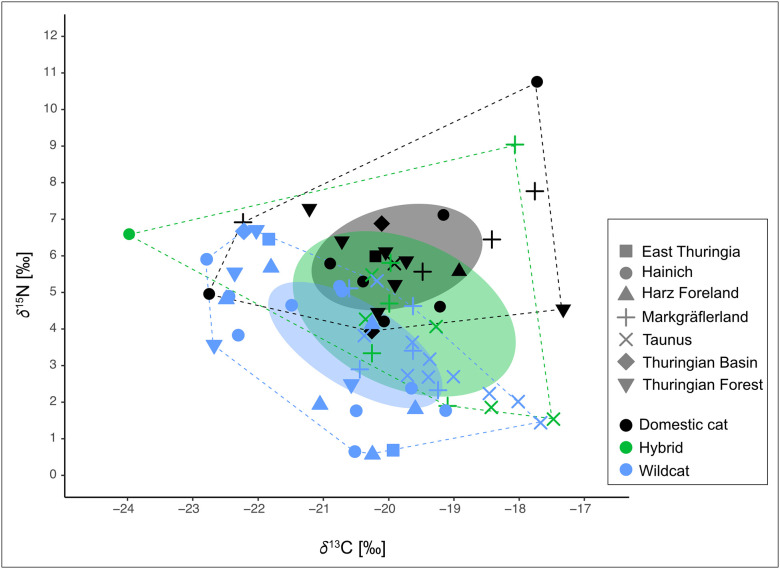
Bivariate plot of δ¹³C and δ¹⁵N values from cat hair, differentiated by taxon (color) and region (symbol shape). Coloured ellipses represent the standard ellipse areas (SEAc, core niche width) for domestic cats (grey/black), hybrids (green), and wildcats (blue). Dotted lines connect the outermost individuals of each group, outlining the Total Area (TA, proxy for total niche space) as a measure of isotopic niche width.

Domestic cats exhibited generally higher *δ*¹⁵N values (6.0 ± 1.5‰) compared to wildcats (3.6 ± 1.7‰), with hybrids showing intermediate values (4.4 ± 2.3‰). Pairwise Wilcoxon tests confirmed significant differences in *δ*¹⁵N between domestic cats and both wildcats (*p* > 0.001) and hybrids (*p* = 0.042), while wildcats and hybrids did not differ significantly (*p* = 0.349). In contrast, *δ*¹³C values were similar across all three groups (domestic cats: −19.8 ± 1.3‰; hybrids: −19.7 ± 1.7‰; wildcats: −20.5 ± 1.4‰; all *p* > 0.05; see Fig 3). For *δ*³⁴S, domestic cats again had the highest values (5.9 ± 1.3‰), hybrids the lowest (3.7 ± 0.7‰), and wildcats fell in between (4.4 ± 1.1‰). The differences were statistically significant between domestic cats and both hybrids (*p* > 0.001) and wildcats (*p* > 0.001), whereas hybrids and wildcats did not differ significantly (*p* = 0.129).

**Fig 4:**
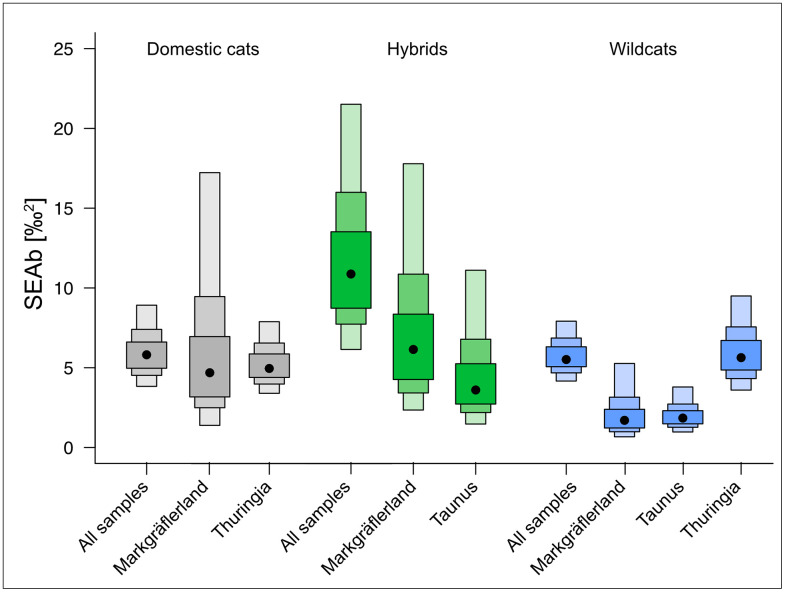
Trophic niche size displayed as Bayesian standard ellipse areas (SEAb) for domestic cats (grey), hybrids (green), and wildcats (blue), shown for all samples and by region. Boxes represent posterior distributions of SEAb; black dots indicate maximum-likelihood SEAc estimates.

**Fig 5:**
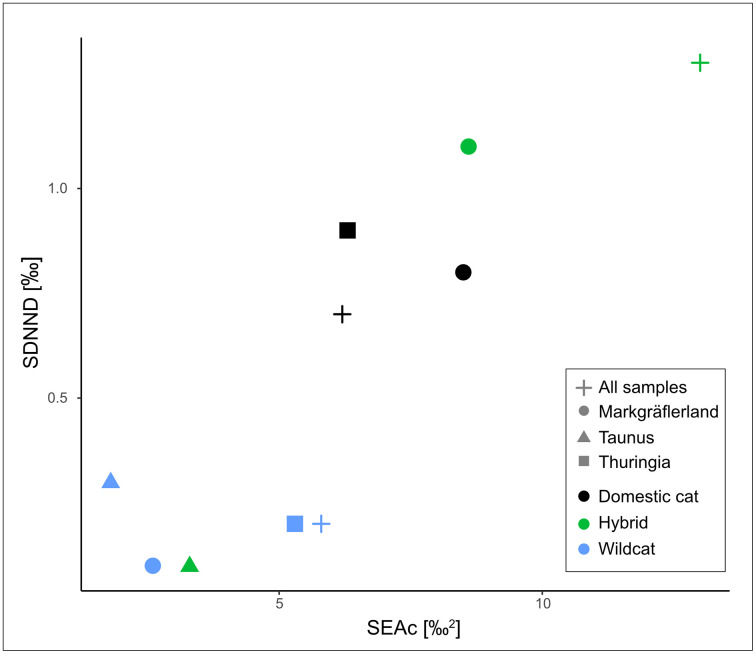
Relationship between corrected standard ellipse area (SEAc) and the standard deviation of nearest neighbour distance (SDNND) across taxa and regions. Symbol shape indicates region; colour indicates taxon.

To explore differences in trophic niche structure between taxa and regions, we calculated a suite of community-wide Layman metrics based on *δ*¹³C and *δ*¹⁵N values (Table 2). These metrics offer complementary insights into the ecological strategies of each group. The total area (TA), defined as the convex hull encompassing all individuals in isotopic space, serves as a proxy for total niche width [52]. Hybrids exhibited the largest TA (25.8‰²), followed by domestic cats (22.4‰²) and wildcats (16.9‰²). To assess core niche width, we calculated corrected standard ellipse areas (SEAc) and Bayesian ellipse areas (SEAb). SEAc approximates the 40% core area of each group’s isotopic distribution and is robust to sample size, while SEAb provides a posterior distribution of ellipse size from Bayesian estimation [53]. In Fig 4, SEAb is visualized as posterior probability distributions (boxes), with the maximum-likelihood SEAc marked by black dots. Consistent with the TA results, hybrids showed the largest core niche widths (SEAc = 12.8‰²), followed by domestic cats (6.1‰²) and wildcats (5.7‰²). Regionally, SEAc values were highest in the Markgräflerland, particularly among hybrids and domestic cats (both 8.4‰²), and lowest in the Taunus and the Markgräflerland wildcats (1.8–2.5‰²), reflecting narrower trophic niches. The *δ*¹³C and *δ*¹⁵N ranges, which reflect variability in basal resources and trophic level respectively [52], followed a similar pattern. Hybrids displayed the largest ranges for both isotopes (6.5‰ and 7.5‰), indicating higher dietary heterogeneity, while wildcats showed more constrained ranges (5.1‰ and 6.1‰). Centroid distance (CD) represents the average distance of individuals from the isotopic centroid and is interpreted as a measure of trophic diversity [52]. Hybrids and wildcats showed higher CD values (2.2 and 2.0) than domestic cats (1.5), with the highest values found in the Markgräflerland and Thuringia regions. The mean nearest neighbour distance (NND) and its standard deviation (SDNND) were used to assess trophic redundancy and evenness, respectively [52]. *Wildcats exhibited the lowest NND (0.4) and SDNND (0.2), indicating high trophic redundancy and an even distribution of individual niches. In contrast, hybrids showed the highest NND (1.3) and SDNND (1.3), reflecting low trophic redundancy, high inter-individual differentiation, and a strongly uneven niche structure.* This relationship is illustrated in Fig 5, where groups with higher SEAc values tend to also exhibit higher SDNND values.

**Table 2:** Summary of Layman metrics calculated from δ¹³C and δ¹⁵N values in cat hair.

Taxon	Region	n	*δ*¹³C range	*δ*¹⁵N range	TA	CD	NND	SDNND	SEAc
Domestic cat	All samples	23	5.4	6.8	22.4	1.5	0.8	0.7	6.1
Hybrid	All samples	11	6.5	7.5	25.8	2.2	1.3	1.3	12.8
Wildcat	All samples	38	5.1	6.1	16.9	2.0	0.4	0.2	5.7
Domestic cat	Markgräflerland	4	4.5	2.2	4.6	1.7	1.8	0.8	8.4
Hybrid	Markgräflerland	5	2.2	7.1	6.8	2.2	1.8	1.1	8.4
Wildcat	Markgräflerland	5	1.4	2.8	2.3	1.1	1.1	0.1	2.5
Hybrid	Taunus	5	2.9	3.9	2.7	1.7	1.1	0.1	3.3
Wildcat	Taunus	10	2.7	3.9	3.0	1.1	0.6	0.4	1.8
Domestic cat	Thuringia	18	5.4	6.8	20.4	1.5	0.9	0.9	6.2
Wildcat	Thuringia	23	3.7	6.1	11.6	2.1	0.4	0.2	5.2

*Metrics include the range in δ¹³C and δ¹⁵N values (proxy for resource and trophic range), the total area (TA) of the convex hull (proxy for total niche space), the mean distance to the centroid (CD; trophic diversity), the mean nearest neighbour distance (NND; trophic redundancy), its standard deviation (SDNND; trophic evenness), and the corrected standard ellipse area (SEAc; core niche width). Values are provided per taxon and region. δ¹³C and δ¹⁵N ranges, CD, NND, and SDNND are given in ‰, TA and SEAc are given in ‰*^*2*^.

To evaluate potential trophic competition, we calculated the extent to which domestic cats and hybrids occupied the isotopic niche of wildcats. Overall, hybrids showed substantial overlap with wildcats, occupying on average 72.6% of the wildcat isotopic niche space. Regionally, this overlap was highest in Markgräflerland (93.0%), followed by Taunus (68.3%). In contrast, domestic cats exhibited minimal niche overlap with wildcats, averaging only 5.9% across all samples. Regional estimates revealed 0.0% overlap in Markgräflerland and 2.6% in Thuringia.

**Fig 6:**
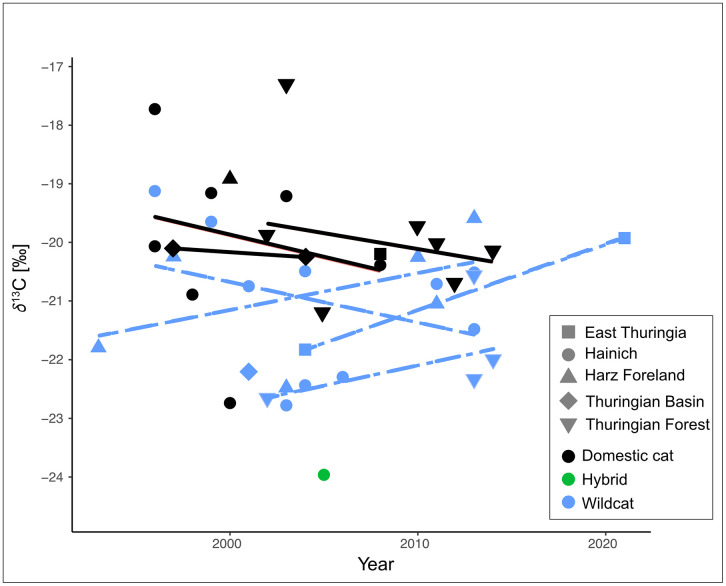
δ¹³C values of wildcat (blue), domestic cat (black), and hybrid (green) hair samples plotted against collection year. Lines indicate robust linear fits per region and taxon. Several regions show weak positive trends in wildcats, suggesting subtle shifts in carbon source use over time.

To investigate potential changes in feeding ecology over time, we examined linear trends in *δ*¹³C, *δ*¹⁵N, and *δ*³⁴S values for wildcats and domestic cats across Thuringian regions. Raw isotope data for all analysed individuals are provided in S1 Table, while S2 Table contains the associated environmental variables used for the Thuringia-specific analyses. Several wildcat populations showed a weak but consistent increase in *δ*¹³C values over time (Fig 6). This was particularly visible in East Thuringia (slope = +0.11), the Thuringian Forest (slope = +0.09, R² = 0.28), and the Harz Foreland (slope = +0.06, R² = 0.23), although none of the trends reached statistical significance. When considering seasonality, the *δ*¹³C increase was mainly driven by summer-grown hair. Stronger positive slopes were observed in summer samples from East Thuringia (+0.11), Hainich (+0.13, R² = 0.33), and the Harz Foreland (+0.26, R² = 0.89), whereas *δ*¹³C values from winter-grown hair remained stable or showed only marginal changes. For instance, in the Hainich region, winter-grown hair even exhibited a weak negative slope (−0.31), despite a positive summer trend. This pattern may reflect seasonal differences in prey availability and habitat use, with winter foraging being more strongly constrained to forest-dominated habitats and less influenced by agriculturally associated prey with elevated *δ*¹³C signatures. The Hainich National Park is characterised by extensive, contiguous deciduous forests, providing a protected environment for wildcats. Such conditions may promote a more forest-bound winter diet and buffer seasonal foraging behaviour from anthropogenic influences. However, given the limited and temporally uneven winter sample size, this interpretation remains tentative and should be treated with caution. Overall, the seasonal patterns support the interpretation that upward trends in *δ*¹³C may reflect subtle dietary shifts during the summer months. In contrast, domestic cats showed variable *δ*¹³C values without clear temporal patterns. For *δ*¹⁵N and *δ*³⁴S, no consistent temporal changes were observed in wildcats. One exception was a significant *δ*³⁴S increase in Harz Foreland wildcats (slope = +0.10, *p* = 0.045, R² = 0.68), which may indicate shifting environmental sulfur baselines.

**Fig 7:**
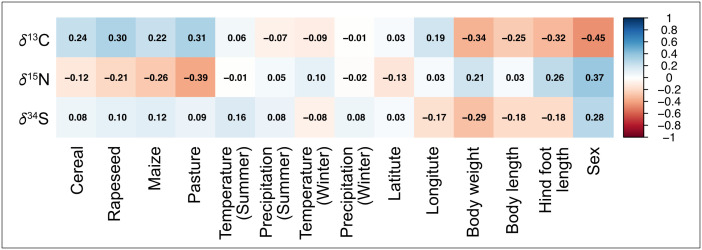
Pearson correlation coefficients (r) between stable isotope values (δ¹³C, δ¹⁵N, δ³⁴S) and environmental (climate, agriculture), geographical, and morphological variables. Positive correlations are shown in blue, negative correlations in red. Only data from wildcats in Case Study 2 (Thuringia) were included.

To further explore ecological and physiological drivers of isotopic variation, we examined Pearson correlations between stable isotope values (*δ*¹³C, *δ*¹⁵N, *δ*³⁴S) and environmental, morphological, and geographical variables (Fig 7). The correlation analysis revealed distinct relationships between isotope values and explanatory variables, especially land use and individual traits. *δ*¹³C values showed moderate positive correlations with pasture (r = 0.31), rapeseed (r = 0.30), cereals (r = 0.24) and maize (r = 0.22), suggesting a dietary contribution from prey linked to agricultural environments. In contrast, *δ*¹³C was negatively correlated with body weight (r = −0.34), body length (r = −0.25), hind foot length (r = −0.32), and sex (r = −0.45). This indicates that smaller individuals and likely females tend to show higher *δ*¹³C values. A corresponding t-test supports this pattern, with females exhibiting significantly higher *δ*¹³C values than males (t(21) = 2.58, *p* = 0.0175). *δ*¹⁵N values, which reflect trophic level, showed positive correlations with body weight (r = 0.21), hind foot length (r = 0.26), and sex (r = 0.37), indicating that males and larger individuals tend to have higher *δ*¹⁵N values. This trend is consistent with a weak but non-significant difference in *δ*¹⁵N values between sexes (t(21) = –1.88, *p* = 0.0736). A detailed correlation plot for male and female individuals is given in S1 Fig. *δ*³⁴S values were only weakly associated with the tested variables; the strongest correlations were observed with summer temperature (r = 0.16) and sex (r = 0.28), suggesting limited influence of either environmental gradients or individual characteristics on sulfur isotope variation. Latitude and longitude showed only weak or negligible correlations with all isotope systems, underlining the importance of land-use patterns and morphology over geographic location in shaping isotopic signatures.

## Discussion

### Trophic ecology *of* wildcats, domestic cats and their hybrids

While based on a somewhat limited set of hair samples, our isotopic results provide nuanced insights into the trophic ecology of European wildcats, domestic cats, and their hybrids. While wildcats generally exhibited the narrowest isotopic niche, suggesting a more specialized diet largely reliant on forest-dwelling small mammals (low *δ*¹³C and *δ*¹⁵N values [54,55]), this pattern was not uniform across space and time. For instance, in the Markgräflerland, wildcats displayed a highly constrained isotopic niche, indicative of strong ecological specialization based on hair samples collected in spring 2021. *However, this constrained niche likely reflects a temporally limited pattern of resource use rather than long-term ecological specialization. As the Markgräflerland population represents a recently recolonized range-edge population, the narrow isotopic niche may capture short-term foraging behaviour under locally abundant and predictable prey conditions during a single season, rather than reduced trophic flexibility.* In contrast, long-term data from Thuringian wildcats revealed a temporal increase in *δ*¹³C values, suggesting a gradual dietary shift from forest-dwelling prey toward species associated with agricultural habitats, such as crop-feeding rodents. This trend, together with the large spatial extent and environmental heterogeneity of habitats within Thuringia, likely resulted in the expanded *δ*¹³C range among wildcats observed here. Nevertheless, this range remained smaller than those observed in hybrids or domestic cats, whose diets are generally broader and more strongly shaped by anthropogenic food sources. This progressive niche expansion mirrors findings from Italy [56], where wildcats increasingly consumed prey associated with human-altered landscapes, and is supported by studies highlighting the species’ adaptability to fragmented or agriculturally influenced habitats [30,57].

Our data on the Thuringian deceased cats also reveal distinct sex- and size-related patterns in wildcat trophic ecology. Females exhibited significantly higher *δ*¹³C values compared to males, and *δ*¹³C values correlated negatively with body weight, total length, and hind-foot length. This indicates that smaller individuals, particularly females, tend to forage more frequently in open or edge habitats, where prey species associated with agricultural or mosaic landscapes are more abundant. Oliveira et al. [58] demonstrated that female wildcats show a stronger selection for high-quality habitats with greater structural heterogeneity and prey availability, such as scrubland–agriculture mosaics. Similarly, Beugin et al. [59] found that females predominantly occupy forest interiors with direct access to field edges, allowing optimal foraging–shelter dynamics. In contrast, males exhibited consistently lower *δ*¹³C values, which we interpret as a stronger reliance on forest-dwelling prey species and a preference for structurally closed habitats. The negative correlation between *δ*¹³C and body size supports this view, suggesting that larger individuals, mainly males, forage predominantly within forest interiors characterized by stable, C₃-based food webs and limited anthropogenic influence. This interpretation aligns with spatial ecology studies indicating that males range widely across forest-dominated territories [58,59]. Additionally, *δ*¹⁵N values were positively correlated with body size, implying that larger individuals, predominantly males, may partially feed on prey from higher trophic levels. This may reflect a shift toward larger prey items or small omnivores and insectivores, including birds, in larger individuals, while smaller wildcats appear to rely more heavily on voles and other low-trophic rodents. These patterns align with general carnivore ecology and the sex-specific foraging strategies commonly observed in solitary predators [60].

### Anthropogenic influence *on* wildcat’s trophic behaviour

Anthropogenic impact, particularly through agriculture and hybridization, emerges as an important driver of trophic dynamics in European wildcats. Hybrids showed substantial isotopic niche overlap with wildcats overall (72.8%), reaching 93% in the Markgräflerland and 68.3% in the Taunus. This pronounced overlap, especially in the Taunus, where genetic studies report only low hybridisation rates (~2%, [7]), indicates that hybrids frequently exploit similar trophic resources as wildcats and cannot be considered trophically distinct. In contrast, domestic cats exhibited minimal overlap, averaging only 5.9%, with 0% overlap in the Markgräflerland and similarly low overlap values observed in the Thuringian reference dataset (2.6%), suggesting limited trophic similarity and competitive pressure. These results align with findings from Germain et al. [61], who reported dietary compositions of hybrids in northeastern France as intermediate between wildcats and domestic cats, with considerable niche overlap toward wildcats. Similar conclusions were reached by Biró et al. [33] using traditional dietary analyses of genetically identified wildcats, hybrids, and feral domestic cats, providing independent support for substantial trophic overlap between wildcats and hybrids. Similarly, Tiesmeyer et al. [9] confirmed regionally variable hybridisation across Europe. Their study highlights the need for regional assessments of ecological consequences, such as niche displacement or functional redundancy. In the Markgräflerland, where hybridisation is frequent (~43%, [13]), hybrids not only overlapped substantially with wildcats but also occupied the largest isotopic niche area, reflecting greater dietary flexibility and ecological integration. *In our dataset, hybrids from the Taunus were predominantly backcrosses to wildcats, but also included one F₁ individual, whereas hybrids from the Markgräflerland consisted of F₁ and F₂ generations.* These differences in hybrid composition may also help to explain the varying degree of trophic overlap observed between regions. In the Taunus, where backcrosses to wildcats dominate, the isotopic niche of hybrids closely mirrors that of wildcats, reflecting similar foraging strategies and resource use. In contrast, in the Markgräflerland, where earlier-generation hybrids and backcrosses to domestic cats occur, hybrids displayed not only the highest overlap with wildcats but also the largest isotopic niche area. This broader niche likely reflects the combined trophic flexibility inherited from both parental lineages and a higher propensity to exploit anthropogenic food sources. Such differences underline that the ecological consequences of hybridisation depend not only on its frequency but also on the generational composition of hybrid cohorts. These patterns underscore the link between hybridisation, landscape modification, and shifts in ecological roles, particularly as wildcats expand into human-altered environments and exhibit more synanthropic behaviour [62].

To further investigate this synanthropic relationship, we examined whether regional agricultural land use could help explain the observed *δ*¹³C trends and isotopic niche dynamics in Thuringian wildcats. In our long-term Thuringian dataset, wildcats showed a gradual increase in δ¹³C values over time, indicating a growing dietary contribution from prey associated with agricultural environments, particularly rapeseed, pasture, and cereal cultivation. Agricultural areas play a dual role in wildcat ecology: on the one hand, they structurally shape the landscape and may provide attractive edge habitats; on the other hand, they compete with structured, undisturbed forests, which are traditionally considered the preferred habitat of European wildcats [3,63]. However, the isotopic niches observed in our data suggest a degree of ecological plasticity, with individuals, particularly during summer, exploiting food resources in open areas with increasing intensity over the years. Our isotopic findings are consistent with recent spatial ecological studies suggesting that European wildcats are increasingly utilizing agriculturally dominated landscapes [10,30,57]. Ruiz-Villar et al. [57] demonstrated that wildcat home range size increases in regions with a high proportion of intensive agriculture and low forest edge density, highlighting the need for larger foraging areas due to reduced prey availability in rather monotonous landscapes. Notably, the presence of heterogeneous features like forest edges buffered the negative effects of agricultural intensification, suggesting that structurally complex agricultural mosaics can still support wildcat populations. Our data align with habitat use patterns described by Jerosch et al.[64], who found that wildcats, particularly females, rely heavily on small-scale shelter structures such as hedgerows, fallow fields, and ecotones to navigate and persist within open landscapes. Their results also indicated seasonal shifts in habitat use, with greater tolerance for open habitats during summer when crops provide temporary cover. Our seasonal isotope data similarly show that *δ*¹³C values were higher in summer than in winter, suggesting increased foraging in open agricultural areas during the growing season. In contrast to the clear patterns observed for *δ*¹³C, the weak correlations observed for *δ*³⁴S across environmental and individual variables further support a cautious interpretation of sulfur isotope data in this study. While *δ*³⁴S has the potential to indicate aquatic or marine-derived food sources or anthropogenic inputs, such signals were not consistently expressed in our dataset. This likely reflects the dominance of terrestrial prey and regionally stable sulfur baselines in the studied systems, rather than an absence of anthropogenic influence per se.

Taken together, these findings strongly indicate adaptive trophic responses of wildcats to anthropogenic landscape change. While such plasticity may facilitate persistence in human-dominated habitats, it may also increases the potential for hybridisation with domestic cats and increasing ecological overlap hybrids, posing long-term challenges for the conservation of the wildcat’s ecological and genetic integrity.

### Retrospective isotopic monitoring *as a* tool *for* carnivore trophic ecology

The combined insights from our two case studies, spanning both spatial contrasts and temporal dynamics, highlight the value of retrospective stable isotope analyses for understanding carnivore trophic ecology in complex and changing landscapes. By integrating isotopic data across taxa, time periods, and environmental gradients, this approach enables the reconstruction of dietary patterns, niche shifts, and individual-level variation that may otherwise remain undetected. Our findings demonstrate how stable isotope data derived from archived or opportunistically collected material can reveal not only ecological differentiation among wildcats, domestic cats, and hybrids, but also track subtle responses of wildcat trophic behaviour to anthropogenic pressures such as hybridisation and agricultural intensification. These applications underscore the potential of retrospective isotopic monitoring as a cost-effective, scalable, and non-invasive tool for advancing carnivore ecology and informing long-term conservation strategies. Retrospective isotopic monitoring enables the reconstruction of ecological responses over time, particularly when direct behavioural or dietary data are unavailable. By analysing stable isotope signatures in archived tissues, such as hair, bone, or teeth, researchers can infer past trophic relationships and habitat use with fine temporal resolution. This approach has already proven useful in a variety of systems: for instance, Turner et al. [65] demonstrated how retrospective isotope data revealed ecosystem responses to hydrological regulation over multiple decades in a riverine food web. Similarly, studies in boreal forests showed that *δ*¹³C and *δ*¹⁵N values in mammal tissues can reliably track shifts in foraging behaviour following habitat disturbance, underscoring the method’s sensitivity to land-use change [66–69]. Our findings confirm that stable isotope signatures in wildcat hair reflect both ecological specialization and increasing anthropogenic integration. Importantly, by combining spatial and temporal datasets, we were able to detect subtle shifts in *δ*¹³C values that would likely be missed in short-term studies. Moreover, the inclusion of *δ*³⁴S adds another layer of resolution by potentially capturing baseline shifts due to atmospheric deposition or fertilizer use, which further enhances the method’s applicability in agricultural landscapes [70]. This aligns with observations by Crawford et al. [69], who emphasized that stable isotope approaches can provide long-term baselines for mammalian ecology and highlight delayed responses to environmental pressures. In this context, isotopic monitoring is particularly relevant for protected and elusive carnivores like the European wildcat, whose behavioural adaptations and ecological roles are increasingly shaped by human activity.

### Limitations and future directions

Despite the valuable insights gained, some limitations of this study should be acknowledged. Sample sizes within the individual taxa and regions were relatively small, particularly for hybrids, which limits the statistical resolution and the detection of subtle trophic or seasonal effects. In addition, uneven sample sizes among regions may influence range-based niche metrics such as total area (TA) and isotopic ranges, which should therefore be interpreted cautiously; accordingly, greater emphasis was placed on core niche (SEAc) and nearest-neighbour metrics that are less sensitive to sample size effects. Moreover, the degree and direction of hybridisation (F₁, F₂, or backcrosses to either wildcats or domestic cats) could not be included in the statistical analyses, as this information was linked to different areas (Taunus vs. Markgräflerland). The dataset also comprised a heterogeneous distribution of sexes and seasons, which may have influenced isotopic variation and limited the comparability among groups. Nevertheless, the results provide a robust first overview of the potential of stable isotope analyses on hair as a tool for nature conservation and ecological monitoring. Future studies should aim for broader seasonal coverage and more balanced sampling designs, as marked seasonal differences in foraging behaviour are likely among wildcats, domestic cats, and hybrids. In addition, the analysis of multiple sections of continuously growing tissues, such as tactile hairs (vibrissae) or sequential hair segments, could provide higher temporal resolution and allow the reconstruction of short-term dietary shifts within individuals. Integrating isotopic, genetic, and spatial data will further enhance our understanding of how hybridisation and anthropogenic landscape change jointly shape the trophic ecology of the European wildcat.

## Materials and methods

A total of 78 cat hair samples were analysed in this study, originating from two independent case studies. Case study 1 included 31 hair samples collected during lure stick monitoring in spring 2021 and was designed to compare two regions (Fig 1A & B): the Taunus and the Markgräflerland. In the Taunus, the sample set consisted of 10 wildcats (5 males, 5 females), 1 domestic cat (female), and 5 hybrids (4 males, 1 female). Hybrids from the Taunus were deliberately targeted and oversampled relative to their known population frequency in order to achieve a sample size comparable to that of the Markgräflerland, thereby enabling balanced isotopic niche analyses across regions. This sampling strategy does not reflect population-level hybridisation rates. In the Markgräflerland, 5 wildcats (4 males, 1 female), 4 domestic cats (3 males, 1 female), and 5 hybrids (3 males, 2 females) were included. Case study 2 comprised 47 hair samples collected from road-killed cats in Thuringia between 1995 and 2021 (Fig. 1C). This dataset included 28 wildcats (13 males, 15 females), 18 domestic cats (15 males, 3 females), and 1 hybrid (female). All individuals were genetically tested and assigned to wildcats, domestic cats, or hybrids using a set of ancestry-informative SNP markers designed for hybrid testing [71]. Genotyping and statistical assignment was done as described in [7] and [9]). DNA extraction and pre-PCR analysis was performed in a laboratory dedicated to the processing of contamination-sensitive noninvasively collected material. *Hybrids from the Taunus were mainly backcrosses to wildcats, with one F₁ individual present, while hybrids from the Markgräflerland comprised F₁ and F₂ generations.* For the single hybrid from Thuringia, no further genetic information was available (S1 Table). Wildcat-like domestic cats included both free-ranging and stray individuals with wild-type coat patterns. For a detailed overview of the samples, see Table 1. Data from Thuringia were included to provide a long-term temporal perspective on wildcat trophic ecology and to place regional isotopic patterns into a broader ecological context. In Tables 1 and 2, Thuringian samples were therefore included in summary statistics and niche metrics for descriptive comparison across datasets. However, due to the different aims of case study 2 and the presence of only a single hybrid individual, Thuringia was not treated as a third spatial comparison region for hybrid niche overlap analyses, which focus on the two regions of case study 1.

For case study 1, surplus hairs from genetic analyses (ranging from 2 to >10 hairs per individual) were provided by the Centre for Wildlife Genetics at the Senckenberg Institute (Gelnhausen) and forwarded to the Biogeology Laboratory at the Department of Geosciences, University of Tübingen/ Senckenberg Centre for Human Evolution and Palaeoenvironment (SHEP) for isotopic analysis. In case study 2, hair samples were taken directly from the pelts of wildcat specimens housed in the collection of the Phyletic Museum, University of Jena. Small tufts of hair were carefully cut from the tail region using clean scissors to avoid cross-contamination.

All hair samples were cleaned in the Biogeology Laboratory using a multi-step solvent protocol to remove surface lipids and environmental contaminants. First, the hairs were immersed in a chloroform–methanol solution (2:1) for 1 hour on a shaker to extract surface lipids. This was followed by a 5-minute acetone treatment in an ultrasonic bath, then a 5-minute rinse in Milli-Q water, also in the ultrasonic bath. The acetone and Milli-Q water steps were then repeated to ensure thorough removal of external residues. After cleaning, all samples were dried at 35 °C for 48 hours. Between 0.15 mg and 0.25 mg of each hair sample was weighed into individual tin capsules. Each capsule was then supplemented with at least three times the respective hair sample weight (0.45–0.75 mg) of tungsten trioxide (WO₃) to support complete combustion, and subsequently sealed for analysis.

### Elemental and isotopic analyses

Elemental and isotopic measurements were carried out at the Geoecology Stable Isotope Platform at the University of Tübingen, using a Vario Isotope Cube elemental analyzer in conjunction with an IsoPrime Vision isotope ratio mass spectrometer (IRMS). We used the international references V-PDB for carbon, atmospheric nitrogen (AIR) for nitrogen, and V-CDT for sulfur isotope ratios to calibrate the measured samples. The international laboratory standards USGS-40 (*δ*^13^C = −26.39 ‰; *δ*^15^N = −4.52‰) and USGS-41a (*δ*^13^C = +36.55‰; *δ*^15^N = +47.55‰) on one hand and IAEA-S1 (*δ*^34^S = −0.30‰), IAEA-S2 (*δ*^34^S = +22.62‰) and IAEA-S3 (*δ*^34^S = −32.49‰) on the other hand, as well as two in-house reference materials (modern collagen of camel: *δ*^13^C = −14.8‰; *δ*^15^N = +8.1‰, *δ*^34^S = +13.63‰, and elk: *δ*^13^C = −23.9‰; *δ*^15^N = +2.6‰, *δ*^34^S = +6.68‰) were used to track device stability, and do drift correction. An analytical error below 0.1‰, 0.2 ‰, and 0.4‰ respectively (1σ) was determined for δ^13^C, δ^15^N, and δ^34^S in all the repeated analyses. The reproducibility error for the amounts of C and N was lower than 1%, and lower than 2% for S.

Atomic elemental ratios were calculated using the following formula:


C/N = (measured carbon content x 14.011)/ (measured nitrogen content x 12.007)

C/S = (measured carbon content x 32.060)/ (measured sulfur content x 12.007)

N/S = (measured nitrogen content x 32.060)/ (measured sulfur content x 14.011)

Atomic C/N ratios between 2.9 and 3.6 are accepted [72], while for atomic C/S ratios values between 300 and 900, and for atomic N/S ratios values between 100 and 300 are tolerated [73].

The isotopic ratios are expressed using the *δ* (delta) value as follows:


$$\delta^{\text{1}\text{3}}\text{C }[‰]\;=\;[{{(}^{\text{13}}\text{C}{/}^{\text{12}}\text{C})}_{\text{sample}}/\;{{(}^{\text{13}}\text{C}{/}^{\text{12}}\text{C})}_{\text{reference}}\;-\;\text{1}]\text{ x 1000}$$

$$\delta^{\text{1}\text{5}}\text{N }[‰]\;=\;[{{(}^{\text{15}}\text{N}{/}^{\text{14}}\text{N})}_{\text{sample}}/\;{{(}^{\text{15}}\text{N}{/}^{\text{14}}\text{N})}_{\text{reference}}-\;\text{1}]\text{ x 1000}$$

$$\delta^{\text{3}\text{4}}\text{S }[‰]\;=\;[({{(}^{\text{34}}\text{S}{/}^{\text{32}}\text{S})}_{\text{sample}}/\;{{(}^{\text{34}}\text{S}{/}^{\text{32}}\text{S})}_{\text{reference}}\;-\;\text{1}]\text{ x 1000}$$

We applied a lipid correction to all measured *δ*¹³C values to exclude the isotopic signature of lipids in the hairs. Because lipids are typically depleted in ¹³C relative to proteins, uncorrected values can bias carbon isotope ratios toward lower *δ*¹³C values [74]. Since no chemical lipid extraction could be performed prior to isotope measurement, *δ*¹³C values were lipid-corrected post hoc using an established equation [75].


$$\delta^{\text{1}\text{3}}{\text{C}}_{\text{lipid}-\text{free}}\;=\;\text{0}.\text{982 x }\delta^{\text{1}\text{3}}{\text{C}}_{\text{measured}}-\;\text{0}.\text{028}$$

This correction accounts for the relationship between lipid content and isotopic composition in mammal hair, ensuring comparability among samples with varying lipid levels. Furthermore, we applied a Suess effect correction to all measured values, based on global atmospheric *δ*¹³C trends (see Scripps CO₂ Program [76]), using pre-industrial conditions as the baseline to ensure comparability among samples [77]. The Suess effect refers to the decline in the atmospheric *δ*¹³C signature caused by large-scale combustion of ¹³C-depleted fossil fuels. Since the onset of the industrial revolution, this process has progressively lowered *δ*¹³C values in atmospheric CO₂ and, consequently, in primary producers and higher trophic levels. Correcting for this effect is essential in stable isotope studies involving modern comparative data, as it enables direct comparison with pre-industrial and archaeological/paleontological samples. Without such a correction, temporal shifts in *δ*¹³C could be misinterpreted as ecological or dietary changes rather than reflecting anthropogenic alterations of the global carbon cycle.

### Statistical analysis

All statistical analyses were performed in R (version 4.5.1; R Core Team) using RStudio (version 2025.05.1 Build 513; Posit Software). The full R script is available on the open online data repository zenodo.org (https://doi.org/10.5281/zenodo.17589796). Graphical preparation of figures was conducted with Affinity Designer 2 (version 2.6; Serif Europe Ltd.).

Stable isotope data (*δ*¹³C, *δ*¹⁵N, *δ*³⁴S) were first quality-checked by excluding samples with atomic C/N ratios outside the range 3.0–4.05, %C values > 50, %N values > 20, or missing regional assignments [44,72]. Cleaned data were used to generate descriptive statistics (mean, SD, min, max) for each taxon and region. Differences in isotopic values between taxa (wildcats, domestic cats, and hybrids) were tested using pairwise Wilcoxon rank-sum tests with Benjamini–Hochberg correction for multiple comparisons. To quantify isotopic niches, we applied the Stable Isotope Bayesian Ellipses in R (SIBER [53]) framework. Bayesian standard ellipse areas (SEAb) were estimated using Markov Chain Monte Carlo simulations (20,000 iterations, 3 chains, burn-in = 1,000, thinning = 10). Maximum-likelihood ellipse metrics were calculated for each group, and niche overlap was quantified using maximum-likelihood overlap functions. In addition, Layman metrics (δ¹³C and δ¹⁵N ranges, total area (TA), centroid distance (CD), mean nearest neighbour distance (NND), SDNND) were calculated to assess trophic niche width and structure [52]. We calculated both the corrected standard ellipse area (SEAc), which represents approximately 40% of the data and describes the core isotopic niche, and the TA, which encompasses all data points including outliers and thus reflects the full extent of niche width [53]. Linear models and robust linear models (rlm) were fitted to estimate temporal slopes of *δ*¹³C, *δ*¹⁵N, and *δ*³⁴S. To assess seasonal differences in isotopic signatures, hair samples were assigned to summer-grown or winter-grown growth periods based on the month of collection. European wildcats and domestic cats undergo two main moulting phases per year, in spring and autumn, and hair isotope values integrate dietary information over several months during growth. Following this biology, samples collected between December and April were classified as *summer-grown hair*, reflecting growth during the preceding spring–summer period, whereas samples collected between May and November were classified as *winter-grown hair*, reflecting growth during autumn and winter. To test for sex-specific and body-size-related differences, two-sample t-tests and regression models were performed. Seasonal differences (summer vs. winter hair growth) were assessed using stratified regression analyses.

Meteorological data (monthly mean temperature, precipitation) were obtained from the German Weather Service (DWD) for 1994–2022 and aggregated at the subregion level. Seasonal subsets (summer: June–August; winter: December–February) were analysed separately. Linear regression models were fitted per subregion to quantify trends. Agricultural land-use data (cereal, maize, rapeseed, pasture area) were extracted from the Thuringian State Office for Statistics. Finally, relationships between isotopic values (*δ*¹³C, *δ*¹⁵N, *δ*³⁴S) and explanatory variables (climate, agricultural land use, sex, body size, age, latitude, longitude) were explored using Pearson correlation analyses and simple linear regression models. Correlation analyses were restricted to wildcats from the Thuringian dataset and were based on pairwise complete observations. Sex was coded as a numeric variable (males = 2, females = 1). No multivariate or mixed-effects models were applied due to limited sample sizes and collinearity among explanatory variables.

## Supporting information

S1 Table Complete table with all analysed samples. (XLSX)

S2 Table Supporting table for Figure 7. (XLSX)

S1 Fig Pearson correlation coefficients (r) between stable isotope values (δ¹³C, δ¹⁵N, δ³⁴S) and environmental (climate, agriculture), geographical, and morphological variables shown separately for female (top panel) and male (bottom panel) European wildcats. Positive correlations are shown in blue, negative correlations in red. Only data from wildcats in Case Study 2 (Thuringia) were included.
(TIFF)

## References

[1] Kitchener AC, Breitenmoser-Würsten C, Eizirik EGA, Werdelin L, Wilting A, et al. A revised taxonomy of the Felidae: The final report of the Cat Classification Task Force of the IUCN Cat Specialist Group. Cat News. 2017;11.

[2] Gerngross P, Ambarli H, Angelici FM, Anile S, Campbell R, Ferreras dAP, et al. Felis silvestris. The IUCN Red List of Threatened Species. 2022:e.T181049859A181050999. 2022. 10.2305/IUCN.UK.2022-1.RLTS.T181049859A181050999.en

[3] Yamaguchi, N., Kitchener, A., Driscoll, C. & Nussberger, B. Felis silvestris. The IUCN Red List of Threatened Species. IUCN. 2014. doi: 10.2305/iucn.uk.2015-2.rlts.t60354712a50652361.en

[4] Nussberger B, Currat M, Quilodran CS, Ponta N, Keller LF. Range expansion as an explanation for introgression in European wildcats. Biological Conservation. 2018;218:49–56. doi: 10.1016/j.biocon.2017.12.009

[5] Say L, Devillard S, Léger F, Pontier D, Ruette S. Distribution and spatial genetic structure of European wildcat in France. Anim Conserv. 2012;15:18–27.

[6] Cushman SA, Kilshaw K, Kaszta Z, Campbell RD, Gaywood M, Macdonald DW. Explaining inter-individual differences in habitat relationships among wildcat hybrids in Scotland. Ecol Modell. 2024;491:110656.

[7] Steyer K, Tiesmeyer A, Muñoz-Fuentes V, Nowak C. Low rates of hybridization between European wildcats and domestic cats in a human-dominated landscape. Ecol Evol. 2018;8(4):2290–304. doi: 10.1002/ece3.3650 29468044 PMC5817136

[8] Matias G, Rosalino LM, Alves PC, Tiesmeyer A, Nowak C, Ramos L. Genetic integrity of European wildcats: variation across biomes mandates geographically tailored conservation strategies. Biol Conserv. 2022;268:109518.

[9] Tiesmeyer A, Ramos L, Manuel Lucas J, Steyer K, Alves PC, Astaras C, et al. Range-wide patterns of human-mediated hybridisation in European wildcats. Conserv Genet. 2020;21(2):247–60. doi: 10.1007/s10592-019-01247-4

[10] Nussberger B, Hertwig ST, Roth T. Monitoring distribution, density and introgression in European wildcats in Switzerland. Biological Conservation. 2023;281:110029. doi: 10.1016/j.biocon.2023.110029

[11] Senn HV, Ghazali M, Kaden J, Barclay D, Harrower B, Campbell RD, et al. Distinguishing the victim from the threat: SNP-based methods reveal the extent of introgressive hybridization between wildcats and domestic cats in Scotland and inform future in situ and ex situ management options for species restoration. Evol Appl. 2018;12(3):399–414. doi: 10.1111/eva.12720 30828363 PMC6383845

[12] Pierpaoli M, Birò ZS, Herrmann M, Hupe K, Fernandes M, Ragni B, et al. Genetic distinction of wildcat (Felis silvestris) populations in Europe, and hybridization with domestic cats in Hungary. Mol Ecol. 2003;12(10):2585–98. doi: 10.1046/j.1365-294x.2003.01939.x 12969463

[13] Streif S, Kögel R, Rolshausen G, Müller M, Nowak C. Sehr hohe Hybridisierungsraten zwischen Wild- und Hauskatzen in Baden-Württemberg – eine Bedrohung für den Erhalt der Art in der Kulturlandschaft?. Wildbiologische Forschungsberichte. 2022. 105–11.

[14] Steyer K, Kraus RHS, Mölich T, Anders O, Cocchiararo B, Frosch C, et al. Large-scale genetic census of an elusive carnivore, the European wildcat (Felis s. silvestris). Conserv Genet. 2016;17(5):1183–99. doi: 10.1007/s10592-016-0853-2

[15] Berteselli GV, Regaiolli B, Normando S, De Mori B, Zaborra CA, Spiezio C. European wildcat and domestic cat: Do they really differ?. Journal of Veterinary Behavior. 2017;22:35–40. doi: 10.1016/j.jveb.2017.09.006

[16] Bastianelli ML, Premier J, Herrmann M, Anile S, Monterroso P, Kuemmerle T, et al. Survival and cause-specific mortality of European wildcat (Felis silvestris) across Europe. Biological Conservation. 2021;261:109239. doi: 10.1016/j.biocon.2021.109239

[17] Stefen C. Does the European wildcat (Felis silvestris) show a change in weight and body size with global warming?. Folia Zoologica. 2015;64(1):65–78. doi: 10.25225/fozo.v64.i1.a8.2015

[18] Klegarth AR. Synanthropy. The International Encyclopedia of Primatology. 2016. 1–5.

[19] Frynta D, Slabova M, Vachova H, Volfova R, Munclinger P. Aggression and commensalism in house mouse: a comparative study across Europe and the Near East. Aggress Behav. 2005;31:283–93.

[20] Puckett EE, Orton D, Munshi-South J. Commensal Rats and Humans: Integrating Rodent Phylogeography and Zooarchaeology to Highlight Connections between Human Societies. Bioessays. 2020;42(5):e1900160. doi: 10.1002/bies.201900160 32173902

[21] Krajcarz M, Krajcarz MT, Baca M, Golubiński M, Bielichová Z, Bulatović J, et al. The history of the domestic cat in Central Europe. Antiquity. 2022;96(390):1628–33. doi: 10.15184/aqy.2022.128

[22] Krajcarz M, Krajcarz MT, Baca M, Baumann C, Van Neer W, Popović D, et al. Ancestors of domestic cats in Neolithic Central Europe: Isotopic evidence of a synanthropic diet. Proc Natl Acad Sci U S A. 2020;117(30):17710–9. doi: 10.1073/pnas.1918884117 32661161 PMC7395498

[23] Krajcarz M, Van Neer W, Krajcarz MT, Popović D, Baca M, De Cupere B, et al. Stable isotopes unveil one millennium of domestic cat paleoecology in Europe. Sci Rep. 2022;12(1):12775. doi: 10.1038/s41598-022-16969-8 35896571 PMC9329303

[24] Krajcarz M, Makowiecki D, Krajcarz MT, Masłowska A, Baca M, Panagiotopoulou H. On the trail of the oldest domestic cat in Poland. An insight from morphometry, ancient DNA and radiocarbon dating. Int J Osteoarchaeol. 2016;26:912–9.

[25] Driscoll CA, Menotti-Raymond M, Roca AL, Hupe K, Johnson WE, Geffen E, et al. The Near Eastern origin of cat domestication. Science. 2007;317(5837):519–23. doi: 10.1126/science.1139518 17600185 PMC5612713

[26] Hu Y, Hu S, Wang W, Wu X, Marshall FB, Chen X, et al. Earliest evidence for commensal processes of cat domestication. Proc Natl Acad Sci U S A. 2014;111(1):116–20. doi: 10.1073/pnas.1311439110 24344279 PMC3890806

[27] Baumann C. The paleo-synanthropic niche: a first attempt to define animal’s adaptation to a human-made micro-environment in the Late Pleistocene. Archaeol Anthropol Sci. 2023;15(5). doi: 10.1007/s12520-023-01764-x

[28] Baumann C, Bocherens H, Drucker DG, Conard NJ. Fox dietary ecology as a tracer of human impact on Pleistocene ecosystems. PLoS One. 2020;15(7):e0235692. doi: 10.1371/journal.pone.0235692 32697783 PMC7375521

[29] Meysel F. Beobachtungen zur Wiederbesiedlung des Hakel durch die Wildkatze. Naturschutz im Land Sachsen-Anhalt. 2009;46:17–24.

[30] Streif S, Kohnen A, Kraft A, Veith S, Wilhelm C, Sandrini M. Die Wildkatze (Felis s. silvestris) in den Rheinauen und am Kaiserstuhl - Raum-Zeit-Verhalten der Wildkatze in einer intensiv genutzten Kulturlandschaft. Freiburg. 2016.

[31] Nussberger B, Barbosa S, Beaumont M, Currat M, Devillard S, Heurich M, et al. A common statement on anthropogenic hybridization of the European wildcat (Felis silvestris). Front Ecol Evol. 2023;11. doi: 10.3389/fevo.2023.1156387

[32] Higashi M, Burns TP, Patten BC. Trophic niches of species and trophic structure of ecosystems: Complementary perspectives through food network unfolding. Journal of Theoretical Biology. 1992;154(1):57–76. doi: 10.1016/s0022-5193(05)80188-2

[33] Biró Zs, Lanszki J, Szemethy L, Heltai M, Randi E. Feeding habits of feral domestic cats (Felis catus), wild cats (Felis silvestris) and their hybrids: trophic niche overlap among cat groups in Hungary. Journal of Zoology. 2005;266(2):187–96. doi: 10.1017/s0952836905006771

[34] Nielsen JM, Clare EL, Hayden B, Brett MT, Kratina P. Diet tracing in ecology: Method comparison and selection. Methods Ecol Evol. 2017;9(2):278–91. doi: 10.1111/2041-210x.12869

[35] Germain E, Benhamou S, Poulle M‐L. Spatio‐temporal sharing between the European wildcat, the domestic cat and their hybrids. Journal of Zoology. 2008;276(2):195–203. doi: 10.1111/j.1469-7998.2008.00479.x

[36] Zimmermann SS, Brockhaus F, Ebert C, Avduli I, Streif S. DNA-Metabarcoding des Mageninhalts von Wildkatzen, Wildkatzenhybriden und verwilderten Hauskatzen. Wildbiologische Forschungsberichte. 2022;4:55–61.

[37] Klare U, Kamler JF, Macdonald DW. A comparison and critique of different scat-analysis methods for determining carnivore diet. Mammal Review. 2011;41(4):294–312. doi: 10.1111/j.1365-2907.2011.00183.x

[38] Marin-Monfort MD, García-Morato S, Olucha R, Yravedra J, Piñeiro A, Barja I, et al. Wildcat scats: Taphonomy of the predator and its micromamal prey. Quaternary Science Reviews. 2019;225:106024. doi: 10.1016/j.quascirev.2019.106024

[39] Széles GL, Purger JJ, Molnár T, Lanszki J. Comparative analysis of the diet of feral and house cats and wildcat in Europe. Mamm Res. 2017;63(1):43–53. doi: 10.1007/s13364-017-0341-1

[40] Boecklen WJ, Yarnes CT, Cook BA, James AC. On the Use of Stable Isotopes in Trophic Ecology. Annu Rev Ecol Evol Syst. 2011;42(1):411–40. doi: 10.1146/annurev-ecolsys-102209-144726

[41] Steyer K, Simon O, Kraus RHS, Haase P, Nowak C. Hair trapping with valerian-treated lure sticks as a tool for genetic wildcat monitoring in low-density habitats. Eur J Wildl Res. 2012;59(1):39–46. doi: 10.1007/s10344-012-0644-0

[42] Drucker DG, Bocherens H, Creighton JD, Roney PJ. Carbon stable isotopes of mammal bones as tracers of canopy development and habitat use in temperate and boreal contexts. Forest canopies: forest production, Ecosystem health, and climate conditions. 2009. 103–9.

[43] Baumann C, Hussain ST, Roblíčková M, Riede F, Mannino MA, Bocherens H. Evidence for hunter-gatherer impacts on raven diet and ecology in the Gravettian of Southern Moravia. Nat Ecol Evol. 2023;7(8):1302–14. doi: 10.1038/s41559-023-02107-8 37349568

[44] O’Regan HJ, Chenery C, Lamb AL, Stevens RE, Rook L, Elton S. Modern macaque dietary heterogeneity assessed using stable isotope analysis of hair and bone. J Hum Evol. 2008;55(4):617–26. doi: 10.1016/j.jhevol.2008.05.001 18599109

[45] Fry B. Stable isotope ecology. Springer. 2006.

[46] Farquhar GD, Ehleringer JR, Hubick KT. Carbon isotope discrimination and photosynthesis. Annu Rev Plant Physiol Plant Mol Biol. 1989;40:503–37.

[47] Parng E, Crumpacker A, Kurle CM. Variation in the stable carbon and nitrogen isotope discrimination factors from diet to fur in four felid species held on different diets. J Mammal. 2014;95(1):151–9. doi: 10.1644/13-mamm-a-014.1

[48] Zazzo A, Monahan FJ, Moloney AP, Green S, Schmidt O. Sulphur isotopes in animal hair track distance to sea. Rapid Commun Mass Spectrom. 2011;25(17):2371–8. doi: 10.1002/rcm.5131 21818798

[49] Rand AJ, Nehlich O. Diet and Sulfur Isotopes. The Encyclopedia of Archaeological Sciences. Wiley. 2018. 1–4. doi: 10.1002/9781119188230.saseas0186

[50] Meckstroth AM, Miles AK, Chandra S. Diets of Introduced Predators Using Stable Isotopes and Stomach Contents. J Wildl Manag. 2007;71(7):2387–92. doi: 10.2193/2005-527

[51] Kays R, Feranec RS. Using Stable Carbon Isotopes to Distinguish Wild from Captive Wolves. Northeastern Naturalist. 2011;18(3):253–64. doi: 10.1656/045.018.0301

[52] Layman CA, Arrington DA, Montaña CG, Post DM. Can stable isotope ratios provide for community-wide measures of trophic structure?. Ecology. 2007;88(1):42–8. doi: 10.1890/0012-9658(2007)88[42:csirpf]2.0.co;2 17489452

[53] Jackson AL, Inger R, Parnell AC, Bearhop S. Comparing isotopic niche widths among and within communities: SIBER - Stable Isotope Bayesian Ellipses in R. Journal of Animal Ecology. 2011;80(3):595–602. doi: 10.1111/j.1365-2656.2011.01806.x 21401589

[54] Bauduin S, Cassaing J, Issam M, Martin C. Interactions between the short-tailed mouse (Mus spretus) and the wood mouse (Apodemus sylvaticus): diet overlap revealed by stable isotopes. Can J Zool. 2013;91(2):102–9. doi: 10.1139/cjz-2012-0286

[55] Balčiauskas L, Skipitytė R, Garbaras A, Stirkė V, Balčiauskienė L, Remeikis V. Stable Isotopes Reveal the Dominant Species to Have the Widest Trophic Niche of Three Syntopic Microtus Voles. Animals (Basel). 2021;11(6):1814. doi: 10.3390/ani11061814 34204576 PMC8233935

[56] Apostolico F, Vercillo F, La Porta G, Ragni B. Long-term changes in diet and trophic niche of the European wildcat (Felis silvestris silvestris) in Italy. Mamm Res. 2015;61(2):109–19. doi: 10.1007/s13364-015-0255-8

[57] Ruiz-Villar H, Bastianelli ML, Heurich M, Anile S, Díaz-Ruiz F, Ferreras P, et al. Agriculture intensity and landscape configuration influence the spatial use of wildcats across Europe. Biological Conservation. 2023;277:109854. doi: 10.1016/j.biocon.2022.109854

[58] Oliveira T, Urra F, López-Martín JM, Ballesteros-Duperón E, Barea-Azcón JM, Moléon M, et al. Females know better: Sex-biased habitat selection by the European wildcat. Ecol Evol. 2018;8(18):9464–77. doi: 10.1002/ece3.4442 30377515 PMC6194279

[59] Beugin M-P, Leblanc G, Queney G, Natoli E, Pontier D. Female in the inside, male in the outside: insights into the spatial organization of a European wildcat population. Conserv Genet. 2016;17(6):1405–15. doi: 10.1007/s10592-016-0871-0

[60] Gittleman JL, Valkenburgh BV. Sexual dimorphism in the canines and skulls of carnivores: effects of size, phylogency, and behavioural ecology. Journal of Zoology. 1997;242(1):97–117. doi: 10.1111/j.1469-7998.1997.tb02932.x

[61] Germain E, Ruette S, Poulle M-L. Likeness between the food habits of European wildcats, domestic cats and their hybrids in France. Mammalian Biology. 2009;74(5):412–7. doi: 10.1016/j.mambio.2009.05.008

[62] Krüger M, Hertwig ST, Jetschke G, Fischer MS. Evaluation of anatomical characters and the question of hybridization with domestic cats in the wildcat population of Thuringia, Germany. Journal of Zoological Systematics and Evolutionary Research. 2009;47(3):268–82. doi: 10.1111/j.1439-0469.2009.00537.x

[63] Feldmann R. Wildkatze – Felis silvestris. Die Säugetiere Westfalens. Münster: Westfälisches Landesmuseum für Naturkunde. 1984. 323–4.

[64] Jerosch S, Kramer-Schadt S, Götz M, Roth M. The importance of small-scale structures in an agriculturally dominated landscape for the European wildcat (Felis silvestris silvestris) in central Europe and implications for its conservation. Journal for Nature Conservation. 2018;41:88–96. doi: 10.1016/j.jnc.2017.11.008

[65] Turner TF, Krabbenhoft TI, Collyer ML, Krabbenhoft CA, Edwards MS, Sharp ZD. Retrospective stable isotope analysis reveals ecosystem responses to river regulation over the last century. Ecology. 2015;96(12):3213–26. doi: 10.1890/14-1666.1 26909427

[66] Sykut M, Pawełczyk S, Borowik T, Pokorny B, Flajšman K, Hunink T, et al. Environmental factors shaping stable isotope signatures of modern red deer (Cervus elaphus) inhabiting various habitats. PLoS One. 2021;16(8):e0255398. doi: 10.1371/journal.pone.0255398 34388162 PMC8362983

[67] Webster SC, Hinton JW, Chamberlain MJ, Murphy JJ, Beasley JC. Land cover and space use influence coyote carnivory: evidence from stable-isotope analysis. PeerJ. 2024;12:e17457. doi: 10.7717/peerj.17457 38854793 PMC11160434

[68] Darling AF, Bayne EM. The potential of stable isotope (δ 13 C, δ 15 N) analyses for measuring foraging behaviour of animals in disturbed boreal forest. Écoscience. 2010;17(1):73–82. doi: 10.2980/17-1-3287

[69] Crawford K, Mcdonald RA, Bearhop S. Applications of stable isotope techniques to the ecology of mammals. Mammal Review. 2008;38(1):87–107. doi: 10.1111/j.1365-2907.2008.00120.x

[70] Georgi M, Voerkelius S, Rossmann A, Graßmann J, Schnitzler WH. Multielement Isotope Ratios of Vegetables from Integrated and Organic Production. Plant Soil. 2005;275(1–2):93–100. doi: 10.1007/s11104-005-0258-3

[71] Nussberger B, Greminger MP, Grossen C, Keller LF, Wandeler P. Development of SNP markers identifying European wildcats, domestic cats, and their admixed progeny. Mol Ecol Resour. 2013;13(3):447–60. doi: 10.1111/1755-0998.12075 23398610

[72] Szpak P, Valenzuela D. Camelid husbandry in the Atacama Desert? A stable isotope study of camelid bone collagen and textiles from the Lluta and Camarones Valleys, northern Chile. PLoS One. 2020;15(3):e0228332. doi: 10.1371/journal.pone.0228332 32160199 PMC7065742

[73] Nehlich O. The application of sulphur isotope analyses in archaeological research: A review. Earth-Science Reviews. 2015;142:1–17. doi: 10.1016/j.earscirev.2014.12.002

[74] Post DM, Layman CA, Arrington DA, Takimoto G, Quattrochi J, Montaña CG. Getting to the fat of the matter: models, methods and assumptions for dealing with lipids in stable isotope analyses. Oecologia. 2007;152(1):179–89. doi: 10.1007/s00442-006-0630-x 17225157

[75] Rioux È, Pelletier F, St‐Laurent M. Influence of lipids on stable isotope ratios in mammal hair: highlighting the importance of validation. Ecosphere. 2019;10(5). doi: 10.1002/ecs2.2723

[76] Scripps CO2 Program. Mauna Loa Observatory, Hawaii and South Pole, Antarctica monthly average δ13C trends. https://scrippsco2.ucsd.edu/assets/graphics/pdf/c13_mlo_spo.pdf?1736161565820. 2025.

[77] Dombrosky J. A ~1000-year 13C Suess correction model for the study of past ecosystems. The Holocene. 2019;30(3):474–8. doi: 10.1177/0959683619887416

