# Supplementary figures and images for: Retrospective isotope monitoring reveals spatial and temporal effects of anthropogenic pressures on the trophic ecology of European wildcats (Felis silvestris) in Germany
Source: PLoS One. 2026 Feb 25;21(2):e0343705. doi: 10.1371/journal.pone.0343705 (PMC12935249; doi:10.1371/journal.pone.0343705)

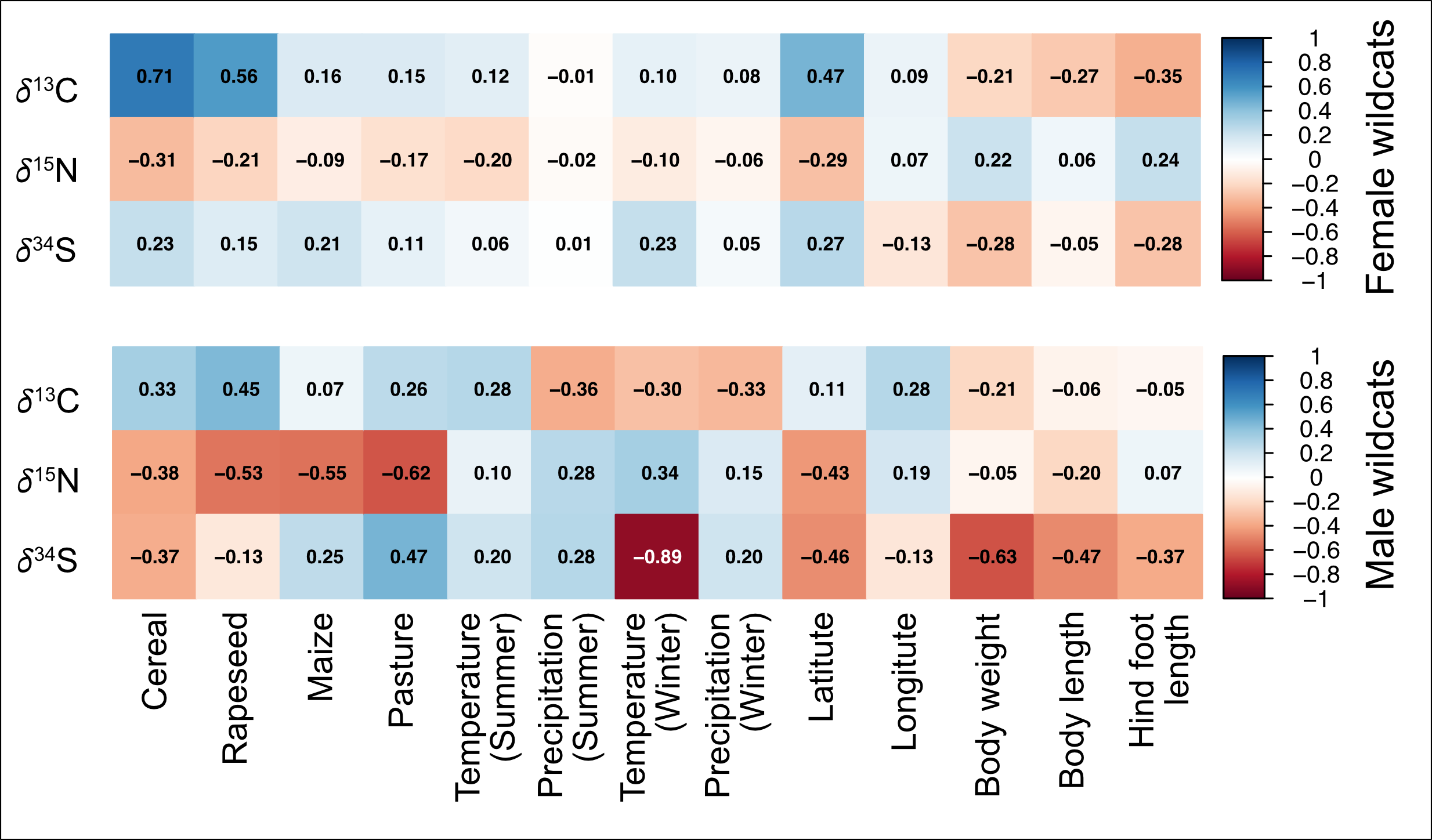

Supplement: S1 Fig — Positive correlations are shown in blue, negative correlations in red. Only data from wildcats in Case Study 2 (Thuringia) were included. (TIFF) [file pone.0343705.s001.tiff]
